# Human cytomegalovirus UL27 is not required for viral replication in human tissue implanted in SCID mice

**DOI:** 10.1186/1743-422X-3-18

**Published:** 2006-03-29

**Authors:** Mark N Prichard, Debra C Quenelle, Deborah J Bidanset, Gloria Komazin, Sunwen Chou, John C Drach, Earl R Kern

**Affiliations:** 1 Department of Pediatrics, University of Alabama School of Medicine, Birmingham AL, USA; 2 Medical and Research Services, VA Medical Center and Oregon Health and Science University, Portland, OR; 3 Department of Biologic and Materials Sciences, University of Michigan, Ann Arbor, MI, USA

## Abstract

Inhibition of the human cytomegalovirus UL97 kinase by maribavir is thought to be responsible for the antiviral activity of this compound. Some mutations that confer resistance to maribavir map to *UL97*, however additional mutations that also confer resistance to the drug were mapped to *UL27*. These open reading frames share a low level of homology, yet the function of pUL27 remains unknown. A recombinant virus with a deletion in the UL27 open reading frame was reported previously to exhibit a slight replication deficit, but a more important function in vivo was hypothesized given its homology to the UL97 kinase. The potential for an important function in vivo was investigated by determining if these knockout viruses could replicate in human tissue implanted in SCID mice. None of the AD169 derived viruses replicated well in the implanted thymus/liver tissue, and is consistent with previous observations, although all of the viruses replicated to some degree in retinal tissue implants. Replication of the parent viruses was observed at 7 days post inoculation, whereas no replication was detected with any of the recombinant viruses with deletions in UL27. By day 14, replication was detected in two of the three knockout viruses and in all of the viruses by day 42. These data are consistent with minimal defects observed in cell culture, but are not consistent with an important role for UL27 in vivo. We conclude that UL27 is not required for viral replication in vivo.

## Findings

Although human cytomegalovirus (HCMV) infections are common in the general population, they can cause significant morbidity and mortality in immunocompromised individuals [12]. Several drugs have been approved to treat these infections, including ganciclovir, foscarnet, and cidofovir, yet the toxicity associated with each of these drugs limits their utility [7], and resistance to these agents can emerge during the lengthy treatment regimens [3]. Additional therapies are clearly required and maribavir is currently being evaluated in clinical trials [6]. This molecule inhibits viral replication [8] through the direct inhibition of the UL97 protein kinase [1] and drug resistant mutations selected in cell culture map to *UL97*. A recombinant virus that does not express this kinase [11] has been shown to be highly resistant to the antiviral effects of the drug in vitro [13]. A recombinant virus that does not express pp65 has also been shown to be somewhat resistant the effects of the drug and is thought to be related to its function together with ppUL97 [10]. Recently, drug resistant mutants were selected in cell culture by two laboratories and the mutations that conferred resistance unexpectedly mapped to the UL27 open reading frame [2,5]. This result was intriguing since *UL27 *was reported to be a paralog of *UL97 *and shares 12% identity at the amino acid level [9], but its function remains undefined. Its role in viral infection is clearly associated with the mechanism of action of maribavir and presumably is related to the UL97 kinase since it is the target of the drug. Drug resistant viruses with point mutations in *UL27 *replicate as well as the parent virus, but recombinant viruses containing large deletions in the open reading frame exhibit a slight, but repeatable growth defect of about half a log in single step growth curves [2]. We hypothesized that defects resulting from the deletion of *UL27 *might be more apparent in vivo and sought to characterize its replication in established models of infection using implanted human tissue in SCID mice.

Three viruses with deletions in *UL27 *were examined. The construction and growth characteristics of T2092-1-1-7 were described previously [2], and this recombinant virus contains a deletion that eliminates the amino terminal 80% of the open reading frame starting just upstream of the start codon (coordinates 33021–34565 in X17403). Two additional viruses were constructed by one of us (GK) in the bacterial artificial chromosome, AD169-BAC. One virus, ΔUL27BAC deletes the amino terminal 90% of *UL27 *and the carboxyl terminal portion of *UL28 *(coordinates 32884–34674) including the entire region deleted in T2092-1-1-7. A second virus, 1/3ΔUL27BAC, contains a mutation that that deletes the central third of *UL27 *without disrupting UL28 (coordinates 33452–34053). To confirm the genomic structure of the engineered genomes, restriction fragments were compared to the AD169-BAC and the predicted changes in fragment sizes were observed. No other alternations in the BAC DNA were apparent. The coordinates of the deletions were also confirmed by PCR and sequencing of the PCR products flanking the deletion region. Both of these viruses were similar to T2092-1-1-7 in that they both exhibited modestly reduced replication in cell culture and attained titers approximately 10-fold lower than the control virus. Since the same phenotype of reduced replication efficiency was observed in all three the recombinant viruses, we conclude that it is likely related to the engineered mutations in *UL27*.

Each of these viruses and the parent viruses from which they were derived, were used to inoculate SCID mice containing implanted retinal tissue or thymus/liver tissue [4]. Briefly, four to eight week old male SCID mice were anesthetized with an i.p. injection of ketamine (100 mg/kg), xylazine (15 mg/kg) and the topical anesthetic proparacaine-HCL (0.5%) was instilled in the eyes. A winged infusion needle containing mechanically dissociated human fetal retinal tissue was then inserted into the nasal sclera and into the anterior chamber. At the temporal side of the anterior chamber approximately 5 μl of tissue were injected and the needle was removed. Using similar procedures, the mice were again anesthetized two to eight weeks after implantation and 10 μl of virus (2000 PFU) were injected into the anterior chamber containing the implant. To monitor viral infection in the retinal implants, animals were euthanized at various times after infection. The eyes were removed, temporarily stored in sterile irrigating balanced salt solution, and homogenized in 1.0 ml MEM containing 10% FBS, 2 mM L-glutamine, 200 U/ml penicillin, 50 μg/ml gentamycin and 3 μg/ml fungizone. The homogenate was centrifuged at 1500 rpm for 15 min at 4°C, and the supernatant was removed and frozen at -70°C until assayed for infectious virus using standard a standard plaque assay. Similar procedures were used for studies using thymus/liver tissue [4].

The AD169 strain of HCMV described in Chou et al. appeared to replicate well in this system and attained titers of 4 log_10_PFU/g by 42 days post inoculation (Table 1). A recombinant virus derived from this strain that contained a large deletion in *UL27 *replicated more slowly and no progeny virus was observed on days 7, 16, and 28. Retinal tissue from animals infected with the parent virus yielded titers of at least 3 log_10_PFU/g at each of these times. This experiment was repeated with a second set of viruses constructed in AD169BAC. The wt virus rescued from the BAC, AD169RV, also replicated well in this model and significant quantities of virus were detected at all time points. Thus, animals infected with both variants of the AD169 strain containing *UL27 *yielded detectable virus by 7 days post infection at titers that generally exceeded 3 log_10_PFU/g. In contrast, neither ΔUL27BAC nor 1/3 ΔUL27BAC were detected in tissue at 7 days post infection, suggesting that their replication was impaired to some degree in this system. But, both these viruses were isolated from animals at 16 and 28 days post infection and contrasted results from T2092-1-1-7 where virus was not observed until day 42 of the experiment. The experiments were also conducted in the thymus/liver model of HCMV infection also in SCID mice. In this model, all strains derived from AD169 replicated poorly and no clear pattern emerged. This is consistent with previous reports describing the limited replication of this strain in this system.

**Table 1:** Replication of UL27 deletion mutants in retinal tissue implants in SCID-hu mice.

Virus	Days post infection			
	
	7	16	28	42
AD169 (ATCC)^a^	3.0 ± 0.58 (4/12)^b^	3.5 ± 1.3 (5/12)	3.7 ± 0.87 (10/12)	4.1 ± 1.1 (7/14)
T2092-1-1-7	0 ± 0 (0/12)	0 ± 0 (0/12)	0 ± 0 (0/12)	3.1 ± 0.17 (4/12)
AD169 RV^c^	2.6 ± 0.17 (4/12)	3.6 ± 1.2 (7/12)	3.1 ± 0.52 (4/12)	3.2 ± 0.74 (3/14)
ΔUL27BAC	0 ± 0 (0/12)	3.6 ± 0.87 (5/12)	3.0 ± 0.45 (5/12)	3.4 ± 0.61 (5/14)
1/3ΔUL27BAC	0 ± 0 (0/12)	2.6 ± 0.21 (2/12)	3.5 ± 0.04 (3/12)	3.6 ± 0.52 (6/14)

a. AD169 (ATCC) is the isogenic strain of AD169 for T2092-1-1-7.

b. Number shown is the average titer in units of log_10_PFU/g of tissue ± the standard deviation with the number of positive animals/the number of inoculated animals shown in parentheses.

c. AD169 RV is the isogenic strain of AD169 for UL27Δ and UL27Δ1/3.

It is unclear whether the apparent differences in replication of the *UL27 *mutants at days 16 and 28 reflect actual differences in the viruses capacity to replicate, or is related to the low number of animals that became infected at these points in the study. The latter is more likely since the deletion in T2092-1-1-7 is entirely contained within the deletion of ΔUL27BAC. What is clear is that all of these viruses replicate to a slightly lesser degree than the parent viruses as evidenced by the uniform absence of replication one week following inoculation. This is also consistent with the slightly impaired replication kinetics observed in cell culture. The data taken together suggest that the deletion of UL27 results in a modest decrease in viral replication in vitro and in vivo. Additional experiments will be required to characterize the function of UL27 and its impact on the antiviral activity of maribavir. These studies may also shed light on the relationship between UL27 and the UL97 kinase.

## Abbreviations

human cytomegalovirus HCMV

wild type *wt*

multiplicity of infection MOI

## Competing interests

The author(s) declare that they have no competing interests.

## Authors' contributions

MNP provided critical intellectual input and analysis of the studies. DQ provided critical intellectual input and conducted some of the animal studies. DJB conducted in the animal experiments SC, GK and JCD provided recombinant viruses and provided critical intellectual input. ERK provided critical intellectual input.
